# Feasibility of an Evidence-Based Weight Loss Intervention for a Faith-Based, Rural, African American Population

**Published:** 2011-10-15

**Authors:** Karen Hye-cheon Kim Yeary, Carol E. Cornell, Page Moore, Zoran Bursac, T. Elaine Prewitt, Delia Smith West, Jerome Turner

**Affiliations:** University of Arkansas for Medical Sciences, Department of Health Behavior and Health Education; University of Arkansas for Medical Sciences, Fay Boozman College of Public Health, Little Rock, Arkansas; University of Arkansas for Medical Sciences, Fay Boozman College of Public Health, Little Rock, Arkansas; University of Arkansas for Medical Sciences, Fay Boozman College of Public Health, Little Rock, Arkansas; University of Arkansas for Medical Sciences, Fay Boozman College of Public Health, Little Rock, Arkansas; University of Arkansas for Medical Sciences, Fay Boozman College of Public Health, Little Rock, Arkansas; Boys, Girls, Adults Community Development Center, Marvell, Arkansas

## Abstract

**Background:**

African Americans and rural residents are disproportionately affected by obesity. Innovative approaches to address obesity that are sensitive to the issues of rural African Americans are needed. Faith-based and community-based participatory approaches show promise for engaging racial/ethnic minorities to change health outcomes, but few faith-based weight loss interventions have used a community-based participatory approach.

**Community Context:**

A faith-based weight loss intervention in the Lower Mississippi Delta arose from a 5-year partnership between academic and community partners representing more than 30 churches and community organizations.

**Methods:**

Community and academic partners translated the 16 core sessions of the Diabetes Prevention Program for rural, church-going African American adults. The feasibility of the lay health advisor–led delivery of the 16-week (January-May 2010), 16-session, adapted intervention was assessed in 26 participants from 3 churches by measuring recruitment, program retention, implementation ease, participant outcomes, and program satisfaction.

**Outcome:**

Twenty-two of 26 participants (85%) provided 16-week follow-up data. Lay health advisors reported that all program components were easy to implement except the self-monitoring component. Participants lost an average of 2.34 kg from baseline to 16-week follow-up, for a mean weight change of −2.7%. Participants reported enjoying the spiritual and group-based aspects of the program and having difficulties with keeping track of foods consumed. The intervention engaged community partners in research, strengthened community-academic partnerships, and built community capacity.

**Interpretation:**

This study demonstrates the feasibility of delivering this adapted intervention by lay leaders through rural churches.

## Background

African Americans (1) and rural residents (2) are disproportionately affected by obesity. To combat health disparities, innovative approaches to address obesity that are sensitive to the issues of rural African Americans are needed. Current approaches acknowledge the potential impact of faith-based interventions (3,4) in rural African American communities. Although faith-based obesity interventions have been implemented with some success (4), studies have focused on urban settings and have not used evidence-based behavioral weight loss methods. A community-based participatory research (CBPR) approach can inform the development of programs for African Americans that incorporate sociocultural factors associated with obesity (5) in the context of addressing health disparities. In a CBPR approach, community and academic partners contribute their insights and strengths to all aspects of the collaborative research process (6). However, few weight loss interventions for racial/ethnic minorities have used CBPR (7). This article describes a CBPR faith-based weight loss intervention developed by a community-academic partnership representing the University of Arkansas for Medical Sciences (UAMS) and more than 30 churches and community organizations in the Arkansas Lower Mississippi Delta (LMD).

## Community Context

The WORD (Wholeness, Oneness, Righteousness, Deliverance) intervention took place in the Arkansas LMD, a rural region bordering the Mississippi River (8). Rates of chronic disease are higher in the LMD than in the rest of the nation (9), and LMD counties have higher rates of obesity than non-LMD counties (10). Moreover, racial/ethnic minorities living in the LMD have a higher prevalence of diabetes, obesity, and high blood pressure compared with whites (10).

The WORD's objective was to adapt an evidence-based weight loss intervention for a faith-based, rural population and to test the feasibility of its delivery by lay leaders to African American adults. Another objective was to use a CBPR approach in developing, implementing, and evaluating the intervention (Figure).

**Figure.**
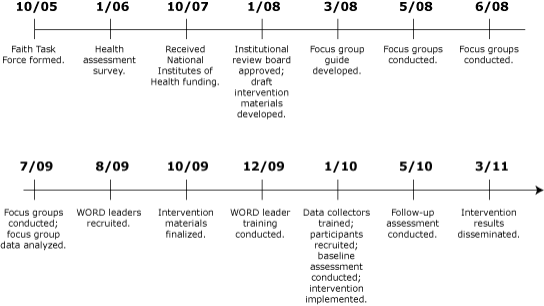
The WORD (Wholeness, Oneness, Righteousness, Deliverance) Intervention Time Line, Arkansas, 2010.

**Table:** 

October 2005: Faith Task Force formed.
January 2006: Health assessment survey.
October 2007: Received National Institutes of Health funding.
January 2008: Institutional review board approved; draft intervention materials developed.
March 2008: Focus group guide developed.
May–July 2008: Focus groups conducted.
July 2009: Focus groups conducted; focus group data analyzed.
August 2009: WORD leaders recruited.
October 2009: Intervention materials finalized.
December 2009: WORD leader training conducted.
January 2010: Data collectors trained; participants recruited; baseline assessment conducted; intervention implemented.
May 2010: Follow-up assessment conducted.
March 2011: Intervention results disseminated.

## Methods

### Community engagement

As an LMD pastor for 17 years, Pastor Jerome Turner had observed the poor health of the communities he served. Karen Hye-cheon Kim Yeary, PhD, approached Pastor Turner in early 2005 to discuss forming partnerships with faith communities to improve health. As a team, Pastor Turner and Dr Yeary met with a group of pastors and interested partners to create the Faith Task Force, which represents more than 30 African American and white LMD churches of various Protestant denominations, local government agencies, community-based organizations, and UAMS. The Faith Task Force is a community-academic partnership that connects faith and health to improve the health of faith communities (Appendix 1).

### The WORD intervention development process

The Faith Task Force engaged a convenience sample of LMD churches to identify health concerns and programs implemented. Participant churches identified physical activity, nutrition, and obesity as primary health concerns. Although few churches had existing health promotion activities, all expressed interest in implementing a health program. On the basis of these findings, the Faith Task Force chose to focus The WORD on obesity and related health behaviors and to adapt the weight loss intervention for African American adults, given the marked racial/ethnic health disparities in the area. The UAMS Institutional Review Board approved the project.

Academic members of the Faith Task Force introduced evidence-based materials from the Diabetes Prevention Program (DPP) (11) and The WORD in North Carolina (7) to develop the intervention. The DPP has produced significant weight loss and health improvements in African American populations (11). Academic and community partners worked together to culturally adapt the DPP curriculum using The WORD in North Carolina. That study identified sociocultural factors related to obesity in rural, church-going African American adults to create and pilot test a culturally appropriate weight loss intervention. The complex interactions between the economic, educational, and historical environment in rural areas, compounded by racial/ethnic disparities, may create a rural culture that reinforces negative health behaviors (2). The WORD in North Carolina identified aspects of rural culture such as the role of social networks and support that facilitate or hinder health behaviors and spiritual ideas such as drawing strength from one's faith to encourage positive health choices.

In adapting the DPP, community members of the Faith Task Force examined materials (eg, faith-based themes, Scripture) and methods (eg, lay health advisor model, group-based format) from The WORD in North Carolina and incorporated culturally appropriate components for their community to produce The WORD. Community partners also provided insider knowledge of the rural African American faith culture to further adapt the intervention, including the addition of Bible studies and further Scriptures. After Faith Task Force members said a broader focus on health would have more community appeal than an exclusive focus on weight loss, the Faith Task Force translated the intervention to emphasize healthy weight and related behaviors to prevent chronic disease.

The intervention was based on social cognitive theory (12) and social support and network models (13). The project placed greater emphasis on social environment dimensions of social cognitive theory (peers, friends, family) than traditional behavioral weight control programs and engaged social relationships by using a lay health advisor model for weight loss promotion. Engaging and building on current social networks through the training of community members was hypothesized to lend to greater cultural sensitivity (ie, spirituality, African American race, rurality). Likewise, the lay health advisor model targets several components of social cognitive theory, including the trained community member as a model for observational learning and the ability of lay health advisors to convey greater salience for behavior change to influence outcome expectations.

### Focus groups

Focus groups were conducted to refine the program materials. Academic partners created an initial draft of the focus-group guide, which contained questions about the materials' clarity, usefulness, appeal, and readability. Community partners then refined the guide.

Community Faith Task Force members led recruitment efforts for focus group participants. Dr Yeary and Pastor Turner co-led 4 groups with pastors, Sunday school teachers, parishioners, and church leaders, in which draft intervention materials were presented and perceptions about the materials solicited.

Dr Yeary used the thematic analysis method to analyze the focus group data. Dr Yeary and Pastor Turner discussed coding decisions and emergent findings until they reached an agreement on common themes and codes (14). To ensure the credibility of inferences, the Faith Task Force reviewed and discussed the findings. Faith Task Force members revised the intervention and curriculum to incorporate the focus group findings and used the refined curriculum to examine the feasibility of delivering this faith-based weight loss program through churches in the Arkansas LMD.

### Intervention feasibility testing

The WORD was conducted in 3 small, rural, African American churches in the LMD. Two churches whose pastors were members of the Faith Task Force volunteered to participate in the pilot intervention. The Faith Task Force recruited 1 additional church through word of mouth. The 3 churches represented 172 members, whose ages ranged from 1 to 91 years. An estimated 135 members met the eligibility criteria (African American, aged ≥21 y, body mass index [BMI] ≥25 kg/m^2^, associated with a participating church, no medical problems that would contraindicate participation, not taking medication that would affect weight loss, and not pregnant or lactating).

After extensive discussion within the Faith Task Force about suitable characteristics for WORD leaders, the pastor of each participating church identified potential candidates and invited them to an informational meeting. The recruitment goal was 6 WORD leaders (2 from each church). WORD leaders received a stipend for delivering the program.

WORD leaders received a 20-hour training that built knowledge about healthy weight, weight-related health behaviors, faith and health, and behavioral strategies through experiential learning. The training also included skills in group facilitation and behavior change promotion. TurningPoint 2008 (Turning Technologies, Youngstown, Ohio) was used to facilitate interactive training and to assess WORD leaders' knowledge and understanding of key concepts. To complete the training, WORD leaders needed to score at least 80% on a final examination.

### Participants

The study goal was to recruit 10 African American adults (BMI ≥25 kg/m²) per church, for a total of 30 participants. WORD leaders in collaboration with the Faith Task Force recruited participants through word of mouth, church announcements, and flyers. The Faith Task Force invited interested participants to an orientation visit, at which eligibility was confirmed and informed consent obtained. Participants received gift cards for completing assessments.

### Intervention implementation and feasibility evaluation

Using the curriculum developed by the Faith Task Force, WORD leaders led small groups of parishioners in 90-minute weekly sessions to address faith and health, healthy eating, physical activity, behavioral strategies to achieve weight control, and overcoming barriers to change. Participants received self-monitoring diaries to record daily dietary intake, physical activity, and time with God between group sessions. WORD leaders reviewed the diaries weekly and returned them with feedback and positive reinforcement.

Dr Yeary trained and certified 4 members of the Faith Task Force to collect self-report data during a 2-day, 8-hour training that incorporated didactic instruction and practice. Data collectors with previous training in the collection of anthropometric data used a Tanita scale (Tanita Corporation, Tokyo, Japan) and a stadiometer to measure the weight and height of all participants.

During a series of meetings, the Faith Task Force discussed which outcomes to assess. Academic partners then created a list of possible domains, offering several scale and item options for domains that rely on self-report measurement. The Faith Task Force collaboratively selected the specific items to include in the evaluation. Trained community data collectors administered measures at baseline and 16-week follow-up that included demographics, body weight, and height to calculate BMI (the primary outcome), and dietary, physical activity, and psychosocial measures. Physical activity was assessed by using a 16-item checklist validated in African Americans. Frequency and duration of different types of activity permitted calculating data from the checklist on metabolic equivalent task (MET) hours per week (15). Percentage of calories from fat was assessed by the National Cancer Institute Quick Food Scan, which reported significant positive correlations between the scan and a 24-hour recall in a multisite community intervention trial that included a site consisting predominately of African Americans (16). Scales developed by Sallis et al (17,18) assessed self-efficacy (18) and social support (17) for healthy dietary and exercise behaviors.

ANOVA models examined the equality of means for baseline demographic, anthropometric, health behavior, and psychosocial variables between the 3 churches. To determine whether means for anthropometric, health behavior, and psychosocial variables differed significantly from baseline to 16-week follow-up, paired *t* tests were used (SAS version 9.1, SAS Institute, Cary, North Carolina). Subgroup analyses examined outcomes among participants who engaged in most of the intervention sessions.

WORD leaders completed a log at each intervention session to record participant attendance and weight. Dr Yeary conducted open-ended interviews with WORD leaders after each intervention session to assess program implementation. Intervention questions asked about inclusion of different components of the intervention (eg, educational component, self-monitoring, group exercise), ease or difficulty in implementing intervention components, and how participants received the intervention. Dr Yeary also observed 6 intervention sessions across the 3 churches and provided constructive feedback to WORD leaders about their delivery. At 16-week follow-up, Dr Yeary conducted semistructured interviews with program participants to assess program satisfaction. These interviews asked participants about their satisfaction with certain program components, aspects of the program they did or did not like, and recommendations for improvement. Indicators of program feasibility included meeting of recruitment goals for WORD leaders and participants, retention of program participants, ease of implementation, significant improvements  in participant variables, and program satisfaction.

## Outcomes

### Intervention development

Focus group participants (n = 36) confirmed the usefulness of linking faith and health through encouraging participants to draw strength from their faith to make positive health changes. Participants liked the inclusion of Scripture from The WORD in North Carolina and proposed additional Scriptures. They requested additional graphics in the materials and identified group exercises feasible for community members (eg, walking). The Faith Task Force used the focus group data to refine the intervention materials.

The collaborative work by the Faith Task Force partners resulted in a 16-week curriculum consisting of adapted materials from the DPP (19) and The WORD in North Carolina (7). Each session included a lesson, a Bible study that connected faith with health, and group exercise. Sessions focused on goal setting and problem solving, with an emphasis on self-monitoring (Appendix 2). The weight goal was 7% reduction of initial body weight, following the DPP. The program also provided DPP dietary goals (25% calories from fat, 1.5 cups fruit/d, 2.5 cups vegetables/d, half of all starches as whole grains) and physical activity targets (150 min/wk) to help achieve the weight loss goal; participants were encouraged to spend time with God at least 15 minutes per day.

### Program feasibility testing

Eleven community residents were recruited and trained to serve as WORD leaders. All were African American women aged 21 or older, and most were current or retired teachers, Sunday school teachers, and Bible study leaders. Seven of 11 recruited people completed the training.

A total of 35 participants were recruited from 12 churches; most (19 of 28 eligible participants) were from the 3 churches that agreed to be a part of the study. Seven recruited participants were ineligible and 2 withdrew before the program began, leaving 26 participants enrolled in the study (Table 1).

No significant differences were noted in demographic and outcome variables of participants between the 3 churches. Retention rates were high; 22 of the 26 enrolled participants provided 16-week follow-up data. Dropouts did not significantly differ from nondropouts in demographics or baseline BMI. On average, 13 participants attended each of the group sessions, and 21 of those enrolled attended at least half of all group sessions (Appendix 2).

Significant differences were reported in means for anthropometric, health behavior, and psychosocial variables from baseline to 16-week follow-up (Table 2). Percentage of initial body weight lost from baseline to follow-up was 2.66% (median, −1.40%; IQR, −6.57% to 0.69%) weight loss among program participants, translating to a mean weight loss of 2.34 kg (median, −1.36 kg; IQR, −4.99 kg to 0.64 kg). Participants significantly increased their total and moderately vigorous physical activity during the intervention period. Although changes in dietary intake were not significant from baseline to 16 weeks, program participants reported increased social support for healthy eating from family and friends and increased social support from family for physical activity.

We examined change over time (from baseline to 16-week follow-up) among participants who attended at least 8 of the 16 group sessions (Table 3) to examine whether more engaged participants (defined as attending at least 8 of 16 group sessions) had greater change in outcomes than those who attended less than 8 of 16 group sessions. Engaged participants lost 4.5% of their initial weight on average and reported significantly more moderately vigorous physical activity, greater encouragement to eat healthfully from family and friends, and greater encouragement to be physically active from family at follow-up. Weight loss averaged 4.04 kg in the engaged group (median, −3.1 kg; IQR, −6.8 kg to 0.05 kg) compared with 0.29 kg in the less engaged group (median, −0.14 kg; IQR, −1.54 to 0.64 kg).

Participants enjoyed the group exercise sessions and the use of Scripture to promote health. Participants identified the group exercises and format of the program as sources of encouragement through providing opportunities for discussion and mutual learning. They reported that the connection between faith and health motivated them to make positive behavior changes because it increased their confidence to make healthy choices, and provided an incentive to do well in the program to show devotion to God.

Many participants said keeping track of their diets entailed too much writing and was overly time-consuming. Some participants did not complete their monitoring books; WORD leaders reported partial or incomplete reporting of foods consumed. Some participants had difficulty reading, writing, and looking up the nutritional content of foods. To encourage self-monitoring, WORD leaders incorporated interactive sessions in which participants brought in food labels and practiced self-monitoring. WORD leaders also met with participants before intervention meetings and facilitated partnerships between participants and those in the congregation with more advanced reading and writing skills. Self-monitoring improved temporarily with these additional efforts, but WORD leaders reported decreased self-monitoring among participants as the program progressed.

## Interpretation

The WORD is one of the few faith-based weight loss interventions to use a CBPR approach. A CBPR approach was useful in recruiting lay health advisors and in culturally adapting the intervention. Community data collectors accurately collected weight and height data but had difficulties collecting survey data, which had some missing data from interviewer error. Hiring data collectors with more experience may be beneficial in a larger trial. Using a CBPR approach also contributed to program sustainability through building community capacity to conduct research; community partners submitted a community grant to continue the program in additional churches.

Study limitations include the use of self-report measures and participant difficulty in self-monitoring, although these limitations would have decreased the magnitude of the intervention's effect. The sample was also a convenience sample.

Lay health advisors were successfully recruited through using a CBPR approach to collaborate with pastors; however, the recruitment goal for eligible participants was not met (goal, 30 participants; recruited, 26). Leading participant recruitment may be too burdensome for lay health advisors, given their other responsibilities. More participants may have been recruited if Faith Task Force community members in addition to lay health advisors led recruitment efforts. The program was feasible to implement with the exception of the self-monitoring component, which required extensive writing. Self-monitoring that does not require writing, such as a checklist format, may produce greater adherence.

Positive changes in weight, physical activity, and social support for health behaviors were consistent in magnitude with other faith-based weight loss programs (7,20). Participants who were more engaged in the program demonstrated greater improvements in weight and physical activity, although even those who participated to a lesser extent showed some improvement. Evidence of The WORD's feasibility in African American adults includes high participant retention, significant changes in program outcomes, and positive participant evaluations of the program. A full-scale controlled trial will be needed to determine if the program was responsible for producing these health improvements. This study demonstrates the feasibility of delivering this adapted intervention by lay leaders through rural churches.

## Figures and Tables

**Table 1.** Participant (n = 26) Characteristics at Baseline, The WORD (Wholeness, Oneness, Righteousness, Deliverance) Intervention, Arkansas, 2010

**Demographic Characteristic**	Mean or n^a^
**Female sex**	22
**Age, y**	50.8
**Employed**	15
**Married**	15
**Education**	
High school education or less	11
Some college	9
College degree or more	6
**Income, $**	
<10,000	7
10,000-29,999	11
≥30,000	8
**Body mass index (kg/m²)**	35.8
**Health behaviors**	
Dietary fat, % kcal	41.8
Total physical activity, METs	24.3
Moderately vigorous recreation, METs	10.8
**Psychosocial variables**	
**Self-efficacy — diet (range, 1-5)**	
Sticking to low-salt, low-fat foods	4.1
Reducing calories	4.3
Reducing salt	4.3
Reducing fat	4.1
**Self-efficacy — physical activity (range, 1-5)**	
Sticking to exercise program	4.1
Making time for physical activity	4.1
**Social support for eating habits — family (range, 5-25)**	
Encouragement	12.1
Discouragement	12.8
**Social support for eating habits — friends (range, 5-25)**	
Encouragement	10.3
Discouragement	13.8
**Social support for physical activity — family**	
Family participation (range, 10-50)	22.0
Family rewards and punishments (range, 3-15)	4.2
**Social support for physical activity — Friends (range, 10-50)**	20.0
Friend participation	22.3

Abbreviations: METs, metabolic equivalents.

**Table 2.** Changes From Baseline to 16-Week Follow-Up, The WORD (Wholeness, Oneness, Righteousness, Deliverance) Intervention, Arkansas, 2010

Variable	16-Week Follow-Up (n = 22)		Change From Baseline (n = 22)		*P* Value^a^

	Mean (SD)	Median (IQR)	**Mean (SD)**	**Median (IQR)**	
**Body weight**					
BMI, kg/m²	35.0 (6.1)	34.6 (4.6)	−0.87 (2.0)	−0.49 (2.3)	.05
Weight, lb	203.7 (40.2)	194.6 (32.4)	−5.15 (12.0)	−3.00 (12.4)	.05
Weight, kg	92.4 (18.2)	88.3 (14.7)	−2.34 (5.5)	−1.36 (5.6)	.05
Weight change, %	NA (baseline)		−2.66 (5.8)	−1.40 (7.3)	.04
**Health behaviors**					
Dietary fat, % kcal	39.8 (8.7)	39.1 (12.7)	−1.2 (12.4)	0.8 (9.7)	.65
Total physical activity, METs	26.8 (13.8)	25.6 (16.9)	5.5 (9.2)	3.5 (17.1)	.01
Moderately vigorous recreation, METs	12.5 (11.0)	9.8 (11.1)	4.2 (7.4)	4.0 (7.9)	.01
**Psychosocial variables**					
**Self-efficacy — diet (range, 1-5)**					
Sticking to low-salt, low-fat foods	3.9 (0.8)	4.1 (1.2)	−0.2 (0.9)	−0.3 (1.2)	.35
Reducing calories	4.2 (0.7)	4.2 (0.8)	−.08 (1.1)	−0.1 (0.8)	.72
Reducing salt	4.3 (0.8)	4.6 (1.2)	.08 (1.3)	0 (0.8)	.77
Reducing fat	4.1 (1.2)	4.3 (1.3)	−0 (1.3)	0 (1.0)	.95
**Self-efficacy — physical activity (range, 1-5)**					
Sticking to exercise program	3.8 (0.86)	4.0 (1.25)	−0.3 (0.93)	−0.2 (1.0)	.19
Making time for physical activity	3.6 (0.87)	3.9 (1.50)	−0.3 (0.96)	−0.2 (1.0)	.19
**Social support for eating habits — family (range, 5-25)**					
Encouragement	17.0 (5.8)	17.5 (9.0)	4.4 (6.2)	3.5 (12.0)	<.001
Discouragement	12.6 (5.6)	11.5 (9.0)	0 (9.1)	0 (13.0)	.99
**Social support for eating habits — friends (range, 5-25)**					
Encouragement	14.3 (5.8)	15.0 (7.0)	3.50 (5.7)	3.5 (7.0)	<.001
Discouragement	13.0 (5.9)	14.0 (12.0)	−1.05 (6.8)	−1.0 (7.0)	.47
**Social support for physical activity — family**					
Family participation (range, 10-50)	26.8 (10.2)	26.7 (16.7)	4.8 (9.4)	5.0 (7.8)	.02
Family rewards and punishments (range, 3-15)	4.4 (2.0)	4.0 (2.0)	.36 (2.1)	0 (1.0)	.42
**Social support for physical activity — friends (range, 10-50)**					
Friend participation	23.3 (9.9)	21.1 (15.6)	1.7 (12.2)	2.8 (15.6)	.51

Abbreviations: SD, standard deviation; IQR, interquartile range; BMI, body mass index; NA, not applicable; METs, metabolic equivalents.

a Calculated from 1-sample *t* test.

**Table 3:** Changes From Baseline to 16-Week Follow-Up (≥50% attendance), The WORD (Wholeness, Oneness, Righteousness, Deliverance) Intervention, Arkansas, 2010

Variable	Baseline (≥50% Attendance) (n = 12)		Follow-Up (≥50% Attendance) (n = 12)		Change From Baseline (≥50% Attendance)		*P* Value^a^

	Mean (SD)	Median (IQR)	Mean (SD)	Median (IQR)	Mean (SD)	Median (IQR)	
**Body weight**							
BMI, kg/m²	35.5 (6.1)	33.9 (9.5)	34.1 (7.2)	31.8 (10.0)	−1.5 (2.3)	−1.2 (2.4)	.05
Weight, lb	214.5 (42.4)	203.2 (42.2)	205.6 (47.9)	191.4 (52.4)	−8.9 (14.1)	−6.9 (14.9)	.05
Weight, kg	97.3 (19.2)	92.2 (19.1)	93.2 (21.7)	86.8 (23.8)	−4.0 (6.4)	−3.1 (6.8)	.05
Weight change, %	NA	NA	NA	NA	−4.5 (6.8)	−4.1 (7.4)	.04
**Health behaviors**							
Dietary fat, % kcal	37.3 (10.2)	33.2 (8.1)	37.0 (7.5)	36.7 (12.4)	−0.3 (12.6)	0.1 (11.0)	.93
Total physical activity, METs	18.3 (10.8)	15.7 (14.0)	23.1 (12.4)	19.8 (18.0)	4.8 (8.8)	1.7 (17.9)	.09
Moderately vigorous recreation, METs	5.94 (6.66)	2.6 (10.2)	13.0 (9.4)	12.0 (8.6)	7.1 (6.5)	5.7 (8.3)	<.001
**Psychosocial variables**							
**Self-efficacy — diet (range, 1-5)**							
Sticking to low-salt, low-fat foods	4.3 (0.7)	4.5 (1.2)	4.1 (0.4)	4.1 (0.4)	−0.2 (0.9)	−0.4 (1.5)	.40
Reducing calories	4.3 (0.9)	4.5 (0.8)	4.2 (0.6)	4.2 (0.8)	0 (1.0)	−0.1 (0.9)	.91
Reducing salt	4.4 (1.0)	5.0 (0.7)	4.4 (0.6)	4.3 (1.2)	−0.1 (1.0)	0 (0.7)	.79
Reducing fat	4.4 (1.2)	5.0 (0.3)	4.4 (0.8)	4.7 (0.8)	0 (1.3)	0 (0.3)	.94
**Self-efficacy — physical activity (range, 1-5)**							
Sticking to exercise program	4.0 (0.8)	4.2 (1.3)	3.8 (0.6)	4.0 (0.9)	−0.2 (1.0)	−0.2 (1.1)	.49
Making time for physical activity	3.9 (0.9)	4.0 (1.2)	3.7 (0.7)	3.8 (1.2)	−0.2 (1.2)	0 (1.4)	.59
**Social support for eating habits — family (range, 5-25)**							
Encouragement	11.5 (4.1)	11.0 (4.0)	16.1 (5.3)	16.0 (7.0)	4.6 (5.7)	4.5 (8.5)	.02
Discouragement	10.7 (4.8)	10.0 (6.5)	12.0 (5.5)	10.5 (9.5)	0.5 (7.8)	0.5 (12.0)	.83
**Social support for eating habits — friends (range, 5-25)**							
Encouragement	9.5 (3.6)	10.5 (7.5)	13.3 (4.0)	14.0 (5.0)	3.8 (4.9)	5.0 (7.5)	.02
Discouragement	14.6 (5.1)	14.0 (5.0)	14.7 (5.7)	15.5 (9.0)	0.1 (5.4)	0 (6.5)	.96
**Social support for physical activity — family**							
Family participation (range, 10-50)	20.4 (8.7)	21.1 (17.2)	25.0 (10.6)	23.9 (18.3)	4.6 (6.5)	3.9 (9.4)	.03
Family rewards and punishments (range, 3-15)	3.6 (1.2)	3.0 (0.5)	4.1 (1.5)	3.5 (1.5)	0.5 (1.2)	0 (0.5)	.17
**Social support for physical activity — friends (range, 10-50)**							
Friend participation	20.4 (10.3)	19.4 (19.4)	21.0 (9.9)	17.8 (15.0)	0.6 (8.6)	1.7 (12.2)	.80

Abbreviations: SD, standard deviation; IQR, interquartile range; BMI, body mass index; NA, not applicable; METs, metabolic equivalents.

a Calculated from 1-sample *t* test.

## Appendices

### Appendix 1. The Community-Based Participatory Research Process of Empowering Communities for Life: Objectives, Methods, and Outcomes

**Table:**

Objectives	Methods	Outcomes
Use a CBPR approach to develop a behavioral intervention	Health assessment survey of churches developed, implemented, and evaluated Focus group guide developed, community partners training in focus group methodology, focus groups implemented and evaluated Translation of evidence-based materials into a culturally appropriate intervention	Engagement of community partners in the research process Health assessment survey development, data collection, and interpretation of results to select a health issue Focus group guide development, recruitment of focus group participants, co-facilitation of focus groups, interpretation of focus group results Using data from the focus groups to refine materials Applying community insider knowledge to increase salience of intervention materials Built community capacity to conduct research Increased understanding and application of survey methodology Increased understanding and application of focus group methodology Increased understanding of the qualitative data analysis process Stronger collaborative relationship between community and academic partners Increased communication between community and academic partners Codevelopment of intervention
Use a CBPR approach to implement a behavioral intervention	Development and implementation of a recruitment strategy for WORD leaders Development and implementation of a recruitment strategy for program participants Provision of ongoing guidance to WORD leaders for the duration of the intervention	Engagement of community partners in the research process Leading recruitment efforts of lay leaders and study participants Participation in maintenance of program fidelity Built community capacity to conduct research Recruitment and retention of study staff and participants Stronger collaborative relationship between community and academic partners Co-implementation of intervention
Use a CBPR approach to evaluate a behavioral intervention	Development and implementation of the assessment instrument Community partners training in data collection	Engagement of community partners in the research process Selection of survey topics to be evaluated, selection of survey items and scales Collection of baseline and follow-up data Built community capacity to conduct research Increased understanding of internal consistency and reliability of survey scales Increased understanding of construct validity Development of skills in survey data collection Stronger collaborative relationship between community and academic partners Codevelopment of the evaluation instrument Community and academic partner worked together to collect data

Abbreviation: CBPR, community-based participatory research; WORD, Wholeness, Oneness, Righteousness, Deliverance Intervention.

### Appendix 2. Weekly Session Topics, The WORD (Wholeness, Oneness, Righteousness, Deliverance) Intervention, Arkansas, 2010

**Table:**

Session	Attendance, n	Topic
1	22	Welcome to The WORD
2	17	Zap the Fat
3	17	Eat Less Fat
4	16	Healthy Eating
5	13	Active in God
6	12	Walk With Him
7	15	Energy In, Energy Out: Calories
8	11	Find the Qs: Cues to Healthy Behaviors
9	10	Break the Chains: Problem Solving
10	10	Eating Out
11	13	Replacing Lies With Truth
12	9	Stopping Slips
13	9	Be HOLY: Remaining Active in God
14	7	Find the Social Qs!
15	8	Managing Stress
16^a^	16	Persevering: Maintaining Healthy Changes

a Session 16 attendance is distinct from completing 16-week follow-up.
